# Response to the letter: role of remote ischemic preconditioning against acute mountain sickness during early phase by Sikri and Chawla

**DOI:** 10.14814/phy2.12498

**Published:** 2015-08-11

**Authors:** Marc M Berger, Hannah Köhne, Lorenz Hotz, Moritz Hammer, Kai Schommer, Peter Bärtsch, Heimo Mairbäurl

**Affiliations:** 1 Department of Anesthesiology, University of HeidelbergHeidelberg, Germany; 2 Department of Anesthesiology, Perioperative and General Critical Care Medicine, Salzburg General Hospital, Paracelsus Medical UniversitySalzburg, Austria; 3 Division of Sports Medicine, Department of Internal Medicine VII, University of HeidelbergHeidelberg, Germany; 4 Member of the German Center for Lung Research (DZL)Germany

We thank Dr. Sikri and Dr. Chawla for their interest in our study. The severity and incidence of AMS were quantified by using the Lake Louise scoring protocol and the AMS-C score of the Environmental Symptom Questionnaire. Subjects were classified as AMS positive with a Lake Louise score ≥5 in combination with an AMS-C score ≥0.70 when headache was present. This approach identifies clinically relevant AMS and increases the specificity in the diagnosis of AMS. Although a Lake Louise score ≥3 points in combination with headache indicates AMS as defined by the Lake Louise Consensus Group (Roach et al. 1993), substantially less than 50% of the mountaineers consider themselves to be sick when fulfilling this criterion score (Bartsch et al. 2004). Applying a Lake Louise score ≥3 points for diagnosing AMS in our study (Berger et al. 2015) increases the incidence from 21% to 57% at 5 h and from 43% to 79% at 18 h in the non-preconditioned control group. In the preconditioned group the incidence would increase from 7% to 29% at 5 h and from 43% to 93% at 18 h, respectively. The differences between the preconditioned and the non-preconditioned group fail statistical significance at both 5 h (*P* = 0.13) and 18 h (*P* = 0.3).

At 8 h remote ischemic preconditioning had no significant effect on the severity of AMS (Lake Louise score: 3.2 ± 0.6 vs. 4.5 ± 0.6, *P* = 0.15; AMS-C score: 0.9 ± 0.2 vs. 1.2 ± 0.2, *P* = 0.14). However, we hesitate to interpret this finding as demonstration for a RIPC-induced biphasic protection. As outlined in the article it is not possible to blind subjects to the application of RIPC. Therefore, we cannot exclude that a placebo effect prevented perception of mild symptoms of AMS in the early hours and caused a delayed onset of AMS after the preconditioning stimulus. Studies lasting longer than 18 h are necessary for testing whether remote preconditioning merely delays the onset of AMS or whether a biphasic pattern with a delayed second protective phase after 24 h as suggested by Bolli (2000) accounts for the observed results.
